# The Abnormal Imaging of SARS-CoV-2: A Predictive Measure of Disease Severity

**DOI:** 10.3389/fmed.2021.694754

**Published:** 2021-10-05

**Authors:** Shangwen Pan, Huaqing Shu, Yaxin Wang, Ruiting Li, Ting Zhou, Yuan Yu, Jiqian Xu, Wei Xiong, Xiaobo Yang, Jiancheng Zhang, Yin Yuan, Shiying Yuan, You Shang

**Affiliations:** Department of Critical Care Medicine, Tongji Medical College, Union Hospital, Huazhong University of Science and Technology, Wuhan, China

**Keywords:** SARS-CoV-2, CT imaging, epidemiological, predictive factors, severity

## Abstract

To investigate the characteristics of SARS-CoV-2 pneumonia and evaluate whether CT scans, especially at a certain CT level, could be used to predict the severity of SARS-CoV-2 pneumonia. In total 118 confirmed patients had been enrolled. All data including epidemiological, clinical characteristics, laboratory results, and images were collected and analyzed when they were administrated for the first time. All patients were divided into two groups. There were 106 severe/critical patients and 12 common ones. A total of 38 of the patients were women. The mean age was 50.5 ± 11.5 years. Overall, 80 patients had a history of exposure. The median time from onset of symptoms to administration was 8.0 days. The main symptoms included fever, cough, anorexia, fatigue, myalgia, headaches, and chills. Lymphocytes and platelets decreased and lactate dehydrogenase increased with increased diseased severity (*P* < 0.05). Calcium and chloride ions were decreased more significantly in severe/critical patients than in common ones (*P* < 0.05). The main comorbidities were diabetes, chronic cardiovascular disease, and chronic pulmonary disease, which occurred in 47 patients. In all 69 patients had respiratory failure, which is the most common SARS-CoV-2 complication, and liver dysfunction presented in 37 patients. Nine patients received mechanical ventilation therapy. One patient received continuous blood purification and extracorporeal membrane oxygenation (EMCO) treatments. The average stay was 18.1 ± 10.8 days. Four patients died. The median of the radiographic score was four in common, and five in the severe/critical illness, which was a significant difference between the two groups. The radiographic score was in negative correlation with OI (ρ = −0.467, *P* < 0.01). The OI in severe/critically ill cases decreased significantly as the disease progressed, which was related to the lesion area in the left lung and right lungs (ρ = 0.688, *R* = 0.733). OI, the lesion area in the left lung and right lungs, lymphocytes, etc. were associated with different degrees of SARS-CoV-2 pneumonia (*P* < 0.05). The lesion area in both lungs were possible predictive factors for severe/critical cases. Patients with SARS-CoV-2 pneumonia showed obvious clinical manifestations and laboratory result changes. Combining clinical features and the quantity of the lesion area in the fourth level of CT could effectively predict severe/critical SARS-CoV-2 cases.

## Introduction

A kind of pneumonia which was caused by severe acute respiratory syndrome coronavirus 2 (SARS-CoV-2) broke out in Wuhan, China at December, 2019 (1–3). At present, three kinds of coronavirus, SARS-CoV, MERS-CoV, and SRAS-CoV-2, have been found to possess the capacity of infecting humans. Although the spread of SARS-CoV-2 pneumonia has been controlled effectively in China, as of 17 December 2020, there have been 74,579,297 patients identified around the world. It has caused tremendous burdens on healthcare and finances. The clinical symptoms of SARS-CoV-2 pneumonia range from mild to critical illness including fever, cough, ARDS (acute respiratory distress syndrome), or even MODS (multiple organ failure), which has caused death in critical patients (4).

The timely diagnosis of SARS-CoV-2 is very important, which not only improves the survival chance of patients but can also help prevent the spread of the disease. Nucleic acid testing is the gold standard for the diagnosis of SARS-CoV-2, which is time-consuming and rigorous. Moreover, the positive incidence of these kits still needs to be improved. All of these disadvantages increase the risk of further spread of the disease. Therefore, as a feasible method, computed tomography (CT) had been used widely in diagnosing SARS-CoV-2 pneumonia (5). However, there are different clinical types of SARS-CoV-2 pneumonia, which require different types of treatment. Currently, doctors determine the severity of SARS-CoV-2 pneumonia and the necessary treatments according to the clinical symptoms and laboratory test results, which are empirical. Therefore, it was urgently required to explore a more objective way to evaluate the severity of the disease. Whether the CT scans could judge the severity of SARS-CoV-2 pneumonia still needed to be explored.

In this study, we investigated 118 patients who had been confirmed with SARS-CoV-2 pneumonia and admitted to Wuhan Jin Yin-tan hospital. We aimed at determining whether a certain level of CT scan could be used to estimate the severity of the disease. The fourth CT section of the lungs was chosen to quantify the lesion area. We found that the lesion area of the lungs was related with Oxygenation index (OI) which was measured by the arterial pressure of oxygen to the fraction of inspired oxygen and had been used as a criterion for ARDS and lung injury, including that caused by SARS-CoV-2 (6, 7). Moreover, the lesion area of the lungs was proved to evaluate the severity of SARS-CoV-2 pneumonia and then guided us to give these patients corresponding treatments, especially severe and critical ones.

## Materials and Methods

The Ethics Commission of Jin Yin-tan hospital approved this study (KY-2020–47.01). Informed consent of this retrospective study was waived due to the segregation policy of the government.

In this study, we retrospectively reviewed patients from December 21 2019 to January 9 2020, who had been diagnosed with SARS-CoV-2 pneumonia, according to the preliminary diagnosis and treatment protocols of the National Health Commission of the People's Republic of China. After administration, all patients underwent laboratory examination to confirm the infection of SARS-CoV-2 and were isolated in hospital for future treatment. We collected all data including epidemiological, clinical characteristics, laboratory results, and imaging findings when they were administrated. Most importantly of all, we divided the patients into two groups (the common group, and the severe/critically ill group) according to the diagnostic criteria of the National Health Commission of the People's Republic of China. For the common group: patients had slight clinical symptoms and some pneumonia presentation in CT scans. For the severe groups, patients who met any of the following conditions were considered: (1) dyspnea, RR ≥ 30 times/minute; (2) resting finger oxygen saturation ≤ 93%; (3) arterial PaO2/FiO2 ≤ 300 mmHg (1 mmHg = 0.133 kPa). High altitude areas over 1,000 m above sea level were corrected for using the equation of PaO2/FiO2 multiplied by atmospheric pressure (mmHg)/760. If the lesion area of pneumonia in CT scans was progressive and ≥50% after 24–48 h, the patient should also be considered as a severe case. For the critically ill groups, patients who met any of the following conditions were considered: (1) respiratory failure requiring mechanical ventilation; (2) shock; (3) any combination of other organ failures which required admission to an intensive care unit.

Chest CT images were obtained from the following scanners: LightSpeed Plus (GE, Medical System, and Milwaukee, USA), Aquilion ONE (Toshiba Medical System, Tokyo, Japan), and UCT 780 (United Imaging, Shanghai, China). A tube voltage of 100 kV or 120 kV and automatic tube current modulation (100–400 mA) were used. Images were reconstructed with a slice thickness of 1.0 or 1.25 mm and an interval of 1.0 or 1.25 mm, respectively. All 118 patients underwent initial CT after the administration.

We still used internationally applicable standard nomenclature about viral pneumonia including ground-glass opacity (GGO), crazy-paving pattern, and consolidation to describe the major CT demonstrations of all patients (8). As a previous study suggested, each of the lobes of the lung were individually analyzed for lesions. We reviewed each of the five lung lobes (the upper and lower lobes of the left lung and upper, middle, and lower lobes of the right lung). The lobes were visually scored from 0 to 1, which means that 0 represented no involvement and 1 represented complete involvement. The total score was the sum of the individual lobar scores with a range of 0–5. The CT were scored by two physician reviewers.

The location of the lesion was recorded and the fourth section of CT scans was chosen to quantitatively estimate the lesion area. The detailed method is as follows: imageLabeler in MATLAB was used to mark and measure the pixel values of the fourth section, which was under 400 in a healthy person. Because of interference derived from the bloodstream in the pulmonary vasculature, we regarded 400 as the threshold of a lesion in the lung, which means that the corresponding linearly threshold of the CT value was −624HU. The areas in which the pixel values were >400 or −624HU were defined as lesion areas. We measured the total area of the left and right lungs, followed by the lesion areas of the left and right lungs, which were used to deduce the proportion of the lesion area.

Statistical analyses were performed using IBM SPSS Statistics Software (version 25; IBM, New York, USA). Continuous variables with a normal distribution were represented by mean ± standard deviation (SD), and medians (interquartile range, IQR) were used for abnormally distributed data. The comparison between groups was performed by *t*-test or non-parametric tests when appropriate. Categorical variables were expressed as a number (%), and the chi-square test was used for comparison between groups. To assess the association between the lesion area of the fourth section and the oxygenation index, Spearman correlation analysis was used. Binary logistic regression analysis was used for predictive factors associated with common, severe, and critical types of pneumonia. All *P*-values were based on a two-tailed test of significance. Statistical significance was always defined as a *P* < 0.05.

## Results

### Demographic, Epidemiologic Characteristics

This single-center, retrospective study was completed in Jin Yin-tan hospital located in Wuhan, China. We reviewed 118 patients who had been diagnosed with COVID-19 pneumonia during the period from December 21, 2019 to January 9, 2020. After administration, we divided all these patients into two groups according to the clinical symptoms and CT. In total 106 patients were confirmed with severe/critical COVID-19 pneumonia. The remaining 12 patients were classified as having common COVID-19 pneumonia. All demographic and epidemiologic characteristics of these patients are shown in Table 1. There were 38 cases in women (32.2%), of whom only two (1.6%) were smokers. The mean age of the patients was 50.5 ± 11.6 years, and 97 of 118 cases (82.2%) were adults aged from 41 to 81 years. All patients lived in Wuhan or around and only seven of them (5.9%) were an aggregative infection. Another 10 of them (8.4%) had been exposed to patients with SARS-CoV-2 infection, or suspected patients. The remaining 70 patients (59.3%) had a history of exposure to the Huanan seafood market. The median time from onset of symptoms to administration was 8.0 (6.0–11.0) days.

**Table 1:** Demographic and epidemiologic characteristics of patients with SARS-CoV-2 pneumonia.

	**Common case**	**Severe/critical illness**	**Total**
	***N* = 12**	***N* = 106**	***N* = 118**
**Sex**			
Female	2 (1.7%)	34 (28.8%)	38 (32.2%)
Male	10 (8.5%)	70 (59.3%)	80 (67.8%)
Age, years	49.8 ± 10.1	50.5 ± 11.7	50.5 ± 11.6
**Exposure**			
Huanan seafood market	11 (9.3%)	59 (50.0%)	70 (59.3%)
Confirmed patient	0 (0.0%)	10 (6.7%)	10 (8.4%)
**Active smoker**			
Yes	0 (0.0%)	2 (1.7%)	2 (1.7%)
No	12 (10.2%)	104 (88.1%)	116 (98.3%)
Median time from onset of symptoms to administration, days	13.0 (7.3–15.8)	8.0 (6.0–10.0)	8.0 (6.0 −11.0)

### Clinical Symptoms and Laboratory Results

The clinical symptoms of these patients, with laboratory examination results, are shown in Tables 2, 3. Generally, the symptoms were fever, cough, anorexia, fatigue, myalgia, headaches, and chills in patients with novel coronavirus pneumonia, of which the incidence was 93.2, 73.7, 61.9, 35.6, 16.9, 12.7, and 10.2%, respectively. Other symptoms included chest pain, nausea, dizziness, pharyngalgia, rhinorrhoea, arthralgia, shiver, palpitation, vomiting, diarrhea, and dyspnea (data are not presented).

**Table 2:** The symptoms of infected patients.

	**Common case**	**Severe/critical illness**	**Total**
	***N* = 12**	***N* = 106**	***N* = 118**
**Symptoms**			
Fever	11 (9.3%)	100 (84.7%)	110 (93.2%)
Cough	8 (6.8%)	79 (66.9%)	87 (73.7%)
Anorexia	4 (3.4%)	69 (58.5%)	73 (61.9%)
Fatigue	4 (3.4%)	37 (31.4%)	42 (35.6%)
Myalgia	3 (2.5%)	17 (14.4%)	20 (16.9%)
Headache	1 (0.8%)	14 (11.9%)	15 (12.7%)
Chills	3 (2.5%)	9 (7.6%)	12 (10.2%)

**Table 3:** The laboratory results of infected patients.

	**Common case**	**Severe/critical illness**	***P* value**
	***N* = 12**	***N* = 106**	
**Blood routine**			
Leucocytes (×10^9^/L; normal range 4–10)	5.1 ± 1.5	5.9 ± 3.3	0.3821
Neutrophils (×10^9^per L; normal range 2–7)	3.2 ± 1.4	4.6 ± 3.3	0.1395
Lymphocytes (×10^9^per L; normal range 0.8–4)	1.5 ± 0.3	1.0 ± 0.5	0.0002
Platelets (×10^9^per L; normal range: male 83–303, female 101-320)	247.7 ± 84.7	188.6 ± 92.7	0.0372
**Blood biochemistry**			
Albumin (g/L; normal range 40–55)	34.2 ± 2.8	33.2 ± 4.0	0.4066
Aspartate aminotransferase (U/L; male normal range 15–40, female 13–35)	33.8 ± 19.7	40.1 ± 23.7	0.371
Alanine aminotransferase (U/L; male normal range 9–50, female 7-40)	49.1 ± 50.8	43.1 ± 44.1	0.6634
Total bilirubin (mmol/L; normal range 0–26)	11.7 ± 7.6	11.4 ± 4.6	0.8369
Serum creatinine (mmol/L; normal range: male 57–97, female 41–73)	71.2 ± 12.4	75.3 ± 21.7	0.521
Lactate dehydrogenase (U/L; normal range 120–250)	228.6 ± 43.0	328.0 ± 110.8	0.0027
Serum calcium (mmol/L; normal range 2.25–2.75)	2.1 ± 0.1	2.0 ± 0.1	0.002
Serum chloridion (mmol/L; normal range 95–100)	106.8 ± 1.9	104.5 ± 3.3	0.0194
**Infection-related biomarkers**			
Procalcitonin (ng/mL; normal range 0.0–0.5)	0.2 ± 0.1	0.2 ± 0.1	0.3962
C-reactive protein (mg/L; normal range 0.0–5.0)	29.6 ± 42.7	66.5 ± 90.4	0.198

In addition, we found that in different degrees of pneumonia patients, the levels of lymphocytes and platelets were decreasing with severity, while lactate dehydrogenase was increasing with increased severity (*P* < 0.05). Calcium and chloride ions in severe/critically ill patients were decreased significantly compared to in commonplace cases ones (*P* < 0.05). There were no significant changes in other results such as leucocytes, neutrophils, procalcitonin, C-reactive protein, and so on.

### Comorbidities, Complications, and Treatments

There were some comorbidities and complications in some of the patients as shown in Table 4. A total of 47 patients (39.8%) had comorbidities, which mainly included diabetes, chronic cardiovascular disease, and chronic pulmonary disease. Other rare comorbidities were malignancy, hepatitis B, myoma of the uterus, and so on (data are not presented). Respiratory failure was the most common complication, occurring in 69 patients. Next most common was liver dysfunction, which presented in 37 patients, followed by acute respiratory distress syndrome (Table 4). Other complications included hypoproteinemia, thrombocytopenia, sepsis, acute kidney injury, and so on (data was not presented).

**Table 4:** Comorbidities and complications of infected patients.

	**Common case**	**Severe/critical illness**	**Total**
	***N* = 12**	***N* = 106**	***N* = 118**
**Comorbidities**			
Diabetes	0 (0.0%)	11 (9.3%)	11 (9.3%)
Chronic cardiovascular disease	3 (2.5%)	6 (5.1%)	9 (7.6%)
Chronic pulmonary disease	0 (0.0%)	4 (3.4%)	4 (3.4%)
**Complication**			
Respiratory failure	2 (1.7%)	67 (56.8%)	69 (58.5%)
Liver dysfunction	4 (3.4%)	34 (28.8%)	38 (32.2%)
Acute respiratory distress syndrome	0 (0.0%)	29 (24.6%)	29 (24.6%)

After administration, all patients were isolated and given supportive and empiric treatments. The general treatments are displayed in Table 5. Oxygen therapy was carried out in most of the patients. A significantly high rate of corticosteroid therapy was recorded in severe/critically ill patients. In total 7 of the 118 patients received non-invasive mechanical ventilation and 2 of the 118 received invasive mechanical ventilation. All nine of these patients were severe/critically ill patients. One patient was treated with continuous blood purification and extracorporeal membrane oxygenation (EMCO) (data was not presented). The average stay was 18.1 ± 10.9 days. After careful treatment, 114 of these patients improved and were discharged.

**Table 5:** Treatments of infected patients.

	**Common case**	**Severe/critical illness**	**Total**
	***N* = 12**	***N* = 106**	***N* = 118**
**Treatments**			
Oxygen therapy	8 (6.8%)	104 (88.1%)	112 (94.9%)
Glucocorticoids	2 (1.7%)	50 (42.4%)	52 (44.1%)
High flow nasal cannula	0 (0.0%)	22 (18.6%)	22 (18.6%)
Immunoglobulin	0 (0.0%)	10 (8.5%)	10 (8.5%)
**Mechanical ventilation**			
Non-invasive	0 (0.0%)	7 (5.9%)	7 (5.9%)
Invasive	0 (0.0%)	2 (1.7%)	2 (1.7%)
**Outcome**			
Death	0 (0.0%)	4 (3.4%)	4 (3.4%)
Improve and Discharge	12 (10.2%)	102 (86.4%)	114 (96.6%)
Average stay, days	9.1 ± 3.1	19.1 ± 11.0	18.1 ± 10.9

### Chest Computed Tomographic Imaging of COVID-19 Pneumonia

**Figure 1:**
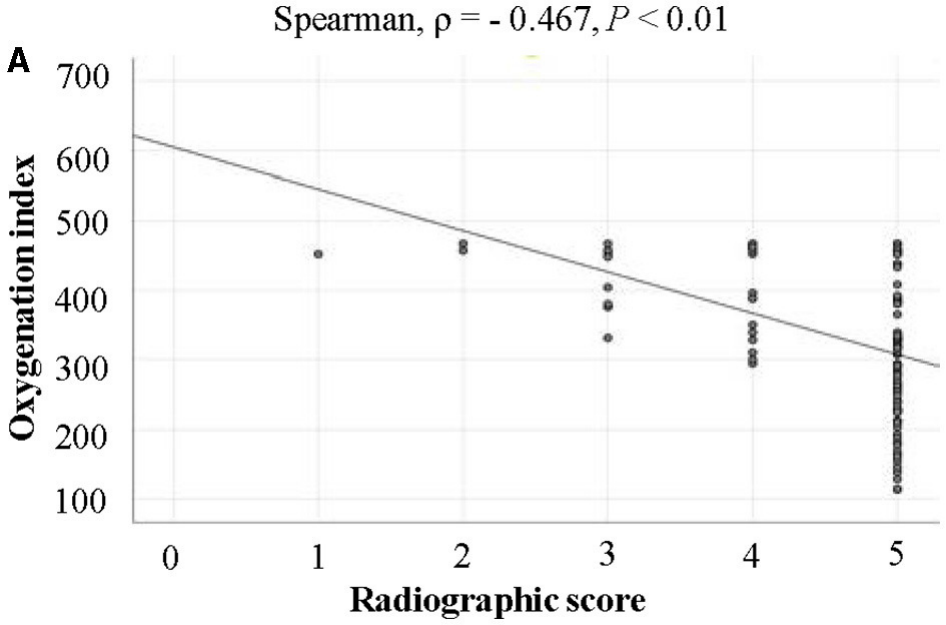
The relationship between the radiographic score and the OI. The radiographic score was significantly negative correlated with the OI.

All infected patients underwent a CT scan at admission in this study; the ground-glass opacities (GGO), fibrotic streaks, and other classical signs of viral pneumonia could be seen in these patients as previously reported. These changes were present in at least one lobe. We reviewed all the CT scans, which found that one patient among the common cases had two affected lobes, three patients with common cases had three lobes affected, six patients with common cases had four affected lobes, and two patients with common cases had five affected lobes. In severe/critically ill patients, one patient had one affected lobe, one patient had two affected lobes, seven patients had three affected lobes, thirteen patients had four affected lobes, and eighty-four patients had five affected lobes. The median of the radiographic score was 4 in common cases, and 5 in severe/critically ill patients, which was a significant difference between the two groups (Supplementary Figure 4). Moreover, radiographic score was also associated with different degrees of novel coronavirus pneumonia (Supplementary Table 1). We reviewed all the OI of these patients. Furthermore, we analyzed the relationship between the radiographic score and the OI, which showed a significantly negative correlation (ρ = −0.467, *P* < 0.01, Figure 1).

### Clinical Predictive Factors for COVID-19 Pneumonia

We reviewed the first CT scans of all patients, and designated the fourth section as the sample section because it most consistently showed obvious abnormalities, and then quantified the lesion area of the fourth section in all patients. Most importantly of all, through Spearman correlation analysis and linear regression analysis, we found that the lesion area in the left lung and right lungs were related to the OI (ρ = 0.688, *R* = 0.733). We further found that the lesion area in both lungs might be used to assess the severity of disease in patients with COVID-19 pneumonia. In univariate analysis, we found that nine risk factors including OI, the lesion area in the left lung, the lesion area in the right lung, and lymphocytes were associated with different degrees of novel coronavirus pneumonia when compared with commonplace ones (*P* < 0.05) (Table 6). Based on these nine variables, we carried out the following multivariate analysis, which showed that the lesion areas in the lungs were possible predictive factors for severe/critical cases (Table 6).

**Table 6:** Univariate and multivariate logistic regression analysis of predictors for severe/critical pneumonia.

**Variable**	**Univariate analysis**			**Multivariate analysis**		
	**OR**	**95% CI**	***P*-value**	**OR**	**95% CI**	***P*-value**
Lymphocytes	0.1	0.025–0.392	0.01			
OI	0.816	0.691–0.963	0.016			
Platelets	0.95	0.989–1.0	0.05			
Creatine kinase	1.017	1.001–1.033	0.035			
Lactate dehydrogenase	1.019	1.006–1.032	0.005			
Serum calcium	0	0.0–0.006	0.001			
Serum chloridion	0.721	0.546–0.952	0.021			
The lesion area in left lung	6.356	2.030–19.899	0.001	6.036	1.554–23.438	0.09
The lesion area in right lung	18.451	2.526–134.785	0.004	10.548	1.301–85.523	0.027

*In all nine risk factors including OI, platelets, creatine kinase, lactate dehydrogenase, serum calcium, serum chloridion, the lesion area in left lung, and the lesion area in right lung, were associated with different degrees of novel coronavirus pneumonia when compared with common ones in univariate analysis. Based on these nine variables, the lesion area in left lung and the lesion area in right lung were the possible predictive factors for the severe/critical ones in multivariate analysis*.

## Discussion

These data provide insights into the evaluation of the severity of patients with COVID-19 associated pneumonia. In line with previous studies, our study confirmed that patients who had been confirmed with SARS-CoV-2 infection had typical CT features (2). Furthermore, we demonstrated a novel and feasible clinical method to assess the severity of infected patients by CT. The lesion area in both the left and right lungs could be used to speculate the severity of SARS-CoV-2 pneumonia.

At the time of this study, SARS-CoV-2 pneumonia was still sweeping across China, with other countries also in danger. As the center of the disease outbreak, we have a profound understanding of clinical presentations and optimal management strategies. Although some specialized treatments have been approved to undergo clinic trials, the main treatments for SARS-CoV-2 patients are still supportive therapies, especially for critical cases, and some patients still inevitably died despite receiving intensive care treatments (4). The potential reason that death still occurred after effective therapies is the lack of simple, reliable methods for severity stratification at onset to guide doctors to better target therapies to patients, especially critically ill ones. Therefore, using readily assessed clinical measures to identify the severity of the SARS-CoV-2 patients and then taking appropriate treatments for each of them is particularly important for the recovery of patients. However, it is difficult to accurately evaluate the severity of patients at present because of a lack of reliable indicators.

ARDS is one of the main complications in SARS-CoV-2 pneumonia, especially in ICU patients (9). Oxygenation index (OI), measured by the arterial pressure of oxygen to the fraction of inspired oxygen, has been used as a criterion for ARDS and lung injury, including that caused by SARS-CoV-2 (6, 7). In our manuscript, we obtained the OI for all patients. We found that with the increase of disease severity, the OI decreased obviously (Supplementary Figure 3). Moreover, in univariate logistic regression analysis, OI was associated with different degrees of novel coronavirus pneumonia when compared with commonplace pneumonia. Radiographic score, which was observed from all five lobes of the patients, and one injured lobe regardless of the size was recorded as 1. The highest radiographic score was 5. We found the median of the radiographic score was 4 in common cases, 5 in severe/critically ill patients. Although there was little numerical difference between the two groups, there was a statistical difference between the two groups (Supplementary Figure 4). Moreover, the radiographic score and the OI were of a significantly negative correlation. Therefore, we analyzed the relationship between radiographic score and different severities of novel coronavirus pneumonia and found that it was associated with different degrees of the disease (Supplementary Table 1). However, the arterial pressure of oxygen measured by arterial blood gas analysis is not available for every patient due to some having no need for blood gas analysis upon admission to hospital, especially the mild cases, and most importantly, many of patients had been treated with oxygen when being initially examined. This urges us to explore more feasible and non-invasive methods to assess the function of the lungs in infected patients, which might guide medical professionals to administer different treatments. Previous studies suggested that these patients had typical chest CT characteristics including ground-glass opacity, fibrotic streaks, and so on (2), which were also presented in our study. As a feasible method, CT has been used widely in diagnosing SARS-CoV-2 pneumonia (5). However, at present, CT is mainly used to qualitatively diagnose, and it is time-consuming waiting for the CT results. Therefore, in this study, we further explore whether CT could be used to estimate the severity of patients with SARS-CoV-2 infection, especially at a certain level. We found that the CT images, with the help of quantitative determination analyses, are associated with the severity of disease in patients with COVID-19 induced pneumonia. It is very important to judge the severity of SARS-CoV-2 pneumonia and then assign patients into different treatment groups, which might administer relevant treatments to these patients, especially for the severely and/or critically ill who need more close attention. This might change the prognosis of these patients. Doctors usually judge the severity of these patients according to the clinical manifestations, which is empirical and appears inaccurate. Therefore, our findings demonstrated the important practical values of CT, which provide an objective indicator to judge the different degrees of patients with COVID-19 associated pneumonia. Rationally applied, CT could estimate risk factors and guide treatments for patients and promote the prognosis of COVID-19 associated pneumonia patients, and for this reason was, we feel, worthy of promotion.

Nonetheless, our study was not without limitations. Firstly, though we have shown enough data to elucidate our conclusion, some laboratory data about patients, especially at the time of admission, may be incomplete or belated. Secondly, this study was by its nature a retrospective investigation, and more studies are still needed. Thirdly, this study was a single-center study and more patients need to be recruited.

In conclusion, we report that in patients infected with COVID-19, the CT scans by quantitative determination analyses could be used to estimate the severity of the disease and guide treatment. This could allow these patients to receive appropriate supportive care, especially in the acute phase, which could increase recovery rates.

## Data Availability Statement

The original contributions presented in the study are included in the article/Supplementary Material, further inquiries can be directed to the corresponding author/s.

## Ethics Statement

The studies involving human participants were reviewed and approved by The Ethics Commission of Jin Yin-tan hospital. Written informed consent for participation was not required for this study in accordance with the national legislation and the institutional requirements. Written informed consent was obtained from the individual(s) for the publication of any potentially identifiable images or data included in this article.

## Author Contributions

SP and HS made equal contributions to this work, performed research, analyzed data, and wrote the manuscript. SY and YS revised and edited the manuscript. All authors read and approved the final manuscript.

## Conflict of Interest

The authors declare that the research was conducted in the absence of any commercial or financial relationships that could be construed as a potential conflict of interest.

## Publisher's Note

All claims expressed in this article are solely those of the authors and do not necessarily represent those of their affiliated organizations, or those of the publisher, the editors and the reviewers. Any product that may be evaluated in this article, or claim that may be made by its manufacturer, is not guaranteed or endorsed by the publisher.
