# Supplementary figures and images for: The Abnormal Imaging of SARS-CoV-2: A Predictive Measure of Disease Severity
Source: Front Med (Lausanne). 2021 Oct 5;8:694754. doi: 10.3389/fmed.2021.694754 (PMC8524080; doi:10.3389/fmed.2021.694754)

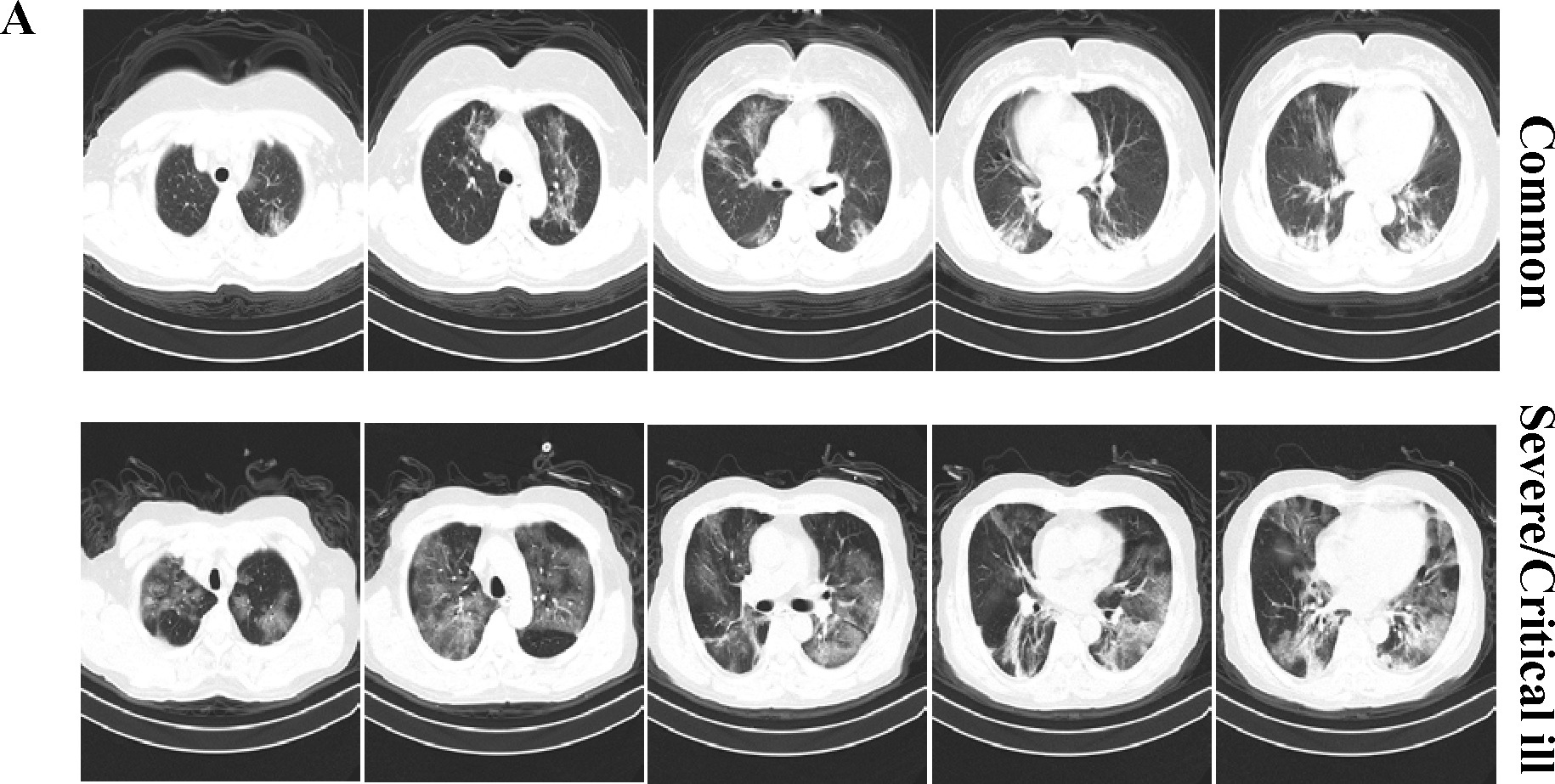

Supplement: Supplementary file 1 [file Image_1.TIF]

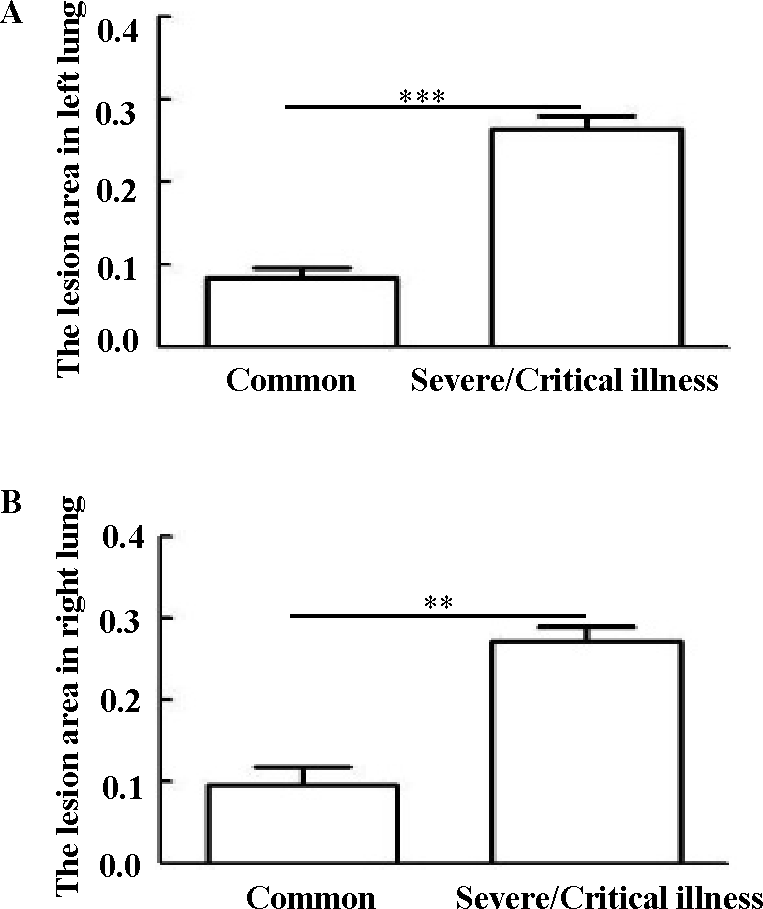

Supplement: Supplementary file 2 [file Image_2.TIF]

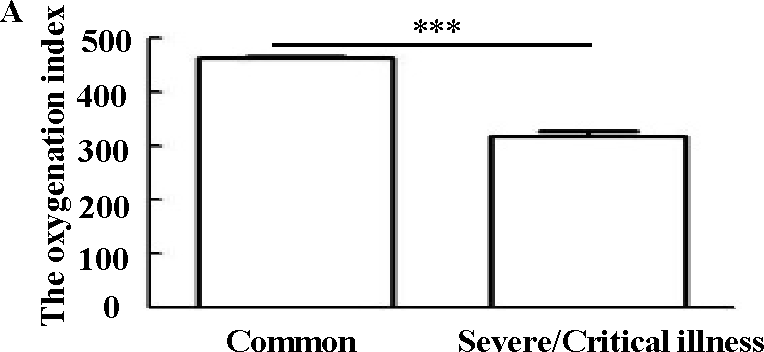

Supplement: Supplementary file 3 [file Image_3.TIF]

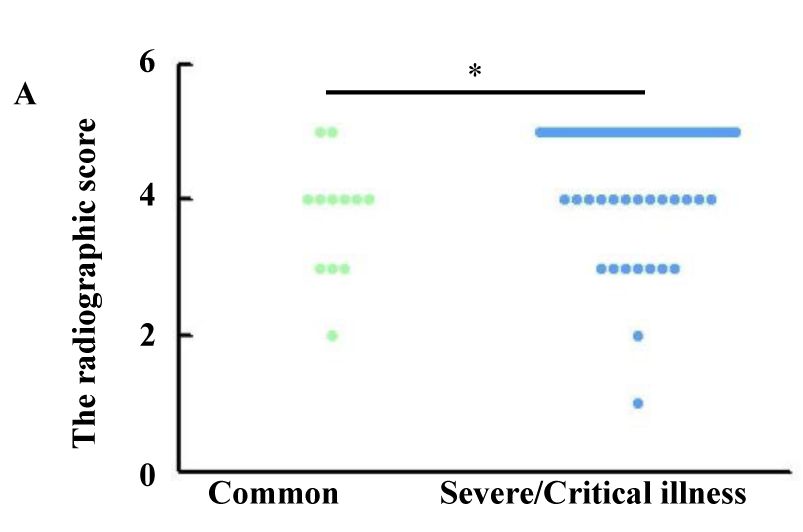

Supplement: Supplementary file 4 [file Image_4.TIF]
