# Supplementary material for: The Abnormal Imaging of SARS-CoV-2: A Predictive Measure of Disease Severity
Source: Front Med (Lausanne). 2021 Oct 5;8:694754. doi: 10.3389/fmed.2021.694754 (PMC8524080; doi:10.3389/fmed.2021.694754)
Supplement: Supplementary file 5 [file Table_1.DOCX]

Supplementary table 1

| Variable | Univariate analysis | | | Multivariate analysis | | |
| --- | --- | --- | --- | --- | --- | --- |
|  | OR | 95%CI | P-value | OR | 95%CI | P-value |
| Lymphocytes | 0.1 | 0.025-0.392 | 0.01 |  |  |  |
| OI | 0.816 | 0.691-0..963 | 0.016 |  |  |  |
| PLT | 0.95 | 0.989-1.0 | 0.05 |  |  |  |
| CK | 1.017 | 1.001-1.033 | 0.035 |  |  |  |
| LDH | 1.019 | 1.006-1.032 | 0.005 |  |  |  |
| Ca | 0.0 | 0.0-0.006 | 0.001 |  |  |  |
| Cl | 0.721 | 0.546-0.952 | 0.021 |  |  |  |
| Radiographic score | 2.793 | 1.499-5.202 | 0.001 | 15.140 | 2.281-100.502 | 0.005 |

Table 1: Univariate and multivariate logistic regression analysis of Radiographic score.
