# Supplementary material for: The Abnormal Imaging of SARS-CoV-2: A Predictive Measure of Disease Severity
Source: Front Med (Lausanne). 2021 Oct 5;8:694754. doi: 10.3389/fmed.2021.694754 (PMC8524080; doi:10.3389/fmed.2021.694754)
Supplement: Supplementary file 6 [file Table_2.DOCX]

**Supplementary Figure**

**Supplementary Fig. 1.** Chest computed tomographic imaging of SARS-CoV-2 infected patients. All patients had CT scans and the typical images including ground-glass opacity could be seen in the lungs.

**Supplementary Fig. 2.** The change of lesion area in different severity of lung. The lesion area in left lung and right lung were increased gradually with the disease getting worse. ^***^P < 0.001 and ^**^P < 0.01 represent Severe /Critical illness vs. Common Case.

**Supplementary Fig. 3.** The OI were decreased gradually with the disease getting worse. ^***^P < 0.001 represent Severe/Critical illness Case vs. Common ones.

**Supplementary Fig. 4.** The radiographic score scatter diagram. *P < 0.05 represent Severe/Critical illness Case vs. Common ones.
